# Tanzawaic Acids, a Chemically Novel Set of Bacterial Conjugation Inhibitors

**DOI:** 10.1371/journal.pone.0148098

**Published:** 2016-01-26

**Authors:** María Getino, Raúl Fernández-López, Carolina Palencia-Gándara, Javier Campos-Gómez, Jose M. Sánchez-López, Marta Martínez, Antonio Fernández, Fernando de la Cruz

**Affiliations:** 1 Instituto de Biomedicina y Biotecnología de Cantabria, Universidad de Cantabria–Consejo Superior de Investigaciones Científicas, Santander, Cantabria, Spain; 2 Biomar Microbial Technologies, Armunia, León, Spain; Centre National de la Recherche Scientifique, Aix-Marseille Université, FRANCE

## Abstract

Bacterial conjugation is the main mechanism for the dissemination of multiple antibiotic resistance in human pathogens. This dissemination could be controlled by molecules that interfere with the conjugation process. A search for conjugation inhibitors among a collection of 1,632 natural compounds, identified tanzawaic acids A and B as best hits. They specially inhibited IncW and IncFII conjugative systems, including plasmids mobilized by them. Plasmids belonging to IncFI, IncI, IncL/M, IncX and IncH incompatibility groups were targeted to a lesser extent, whereas IncN and IncP plasmids were unaffected. Tanzawaic acids showed reduced toxicity in bacterial, fungal or human cells, when compared to synthetic conjugation inhibitors, opening the possibility of their deployment in complex environments, including natural settings relevant for antibiotic resistance dissemination.

## Introduction

Infections due to antibiotic-resistant (AbR) enterobacteria are a worldwide cause of morbidity and mortality [1]. Moreover, the interest in developing new antibiotics by the pharmaceutical industry is declining due to high development costs and the ability of bacteria to evolve quickly and thus overcome antibiotic action [2]. As AbR genes disseminate mostly by conjugation [3, 4], we proposed a new strategy to control AbR dissemination before infection, targeting AbR plasmid conjugation [5, 6]. Efforts to control conjugation include either targeting specific components [7–9] or the overall conjugation process [6, 10]. However, only unsaturated fatty acids (uFAs) were considered effective compounds in practice to inhibit plasmid conjugation in enterobacteria [6, 10]. Bisphosphonates, on the other hand, were recently revealed as nonspecific chelating agents [11] instead of specific inhibitors of plasmid F relaxase [7].

Among previously discovered conjugation inhibitors (COINs), the most potent to date, dehydrocrepenynic acid [6], is extracted from tropical plant seeds [12]. uFAs, such as oleic and linoleic acids, have double bonds susceptible to oxidation [13]. Although triple-bonded fatty acids 2-hexadecynoic acid (2-HDA) and 2-octadecynoic acid (2-ODA) are promising COINs, easily synthesized [14–16] and capable of preventing plasmid invasiveness in a bacterial population [10], they have toxicity issues that must be overcome. Although 2-HDA showed no toxicity in *Escherichia coli*, it was found to be toxic for fungi [17, 18], protozoa [14, 15], gram positive bacteria, some gram-negative bacteria and eukaryotic cells [16, 19, 20]. Because COINs act as prophylactic molecules, but do not elicit a direct therapeutically action, their practical application requires administration in environmental settings where plasmid conjugation occurs. Thus, COIN toxicity must be reduced, ideally completely eliminated, while chemical stability has to be maintained.

We decided to screen AQUAc, a collection of bioactive compounds isolated from aquatic microorganisms, in a search for better COINs. We expected to find compounds with different target specificity, better potency and stability, or less toxic to different cell types. The compound collection was tested by using a whole-cell automated assay. As a result, tanzawaic acids (TZAs) A and B were discovered as natural COINs with reduced toxicity compared to synthetic ones, able to inhibit bacterial conjugation of an important fraction of relevant plasmid groups.

## Results

### High-throughput conjugation (HTC) screening of AQUAc collection

A total of 1,632 partially purified natural compounds extracted from a diversity of marine microorganisms (mainly actinomycetes, fungi and micro-algae) constitute the AQUAc collection from Biomar Microbial Technologies. It contains a high percentage of novel chemical structures (http://www.biomarmicrobialtechnologies.com). The AQUAc collection was analyzed using a luminescence-based HTC screening assay [6]. The IncW plasmid R388 was selected as the test plasmid due to its simple genetic organization [21] and its widespread mating pair formation (MPF) system, similar to that of the well-known *Agrobacterium tumefaciens* Ti plasmid [22]. A total of 9 compounds showed luminescence values under the selected threshold at tested concentrations and were chosen as best hits (S1 Fig). Control assays were carried out to discard hits affecting bacterial growth, plasmid stability, *lux* expression or light production. None of the selected compounds (except perhaps P515) reduced luminescence of control cells containing plasmid pSU2007::Tn*lux*, which emits light constitutively (S2 Fig). Potency assays were subsequently carried out to select the most effective COINs (S1 Table). Two promising hits, P515 and P605, were selected for further analysis. Confirmation of COIN activity by plate-conjugation assays carried out in triplicate (at 50 μg/ml COIN concentration) resulted in relative frequency values of 1% for compound P515 and 20% for compound P605, respectively. Scale-up fermentations of the appropriate organisms were performed, bulk harvested biomass was extracted and serial HTC-guided fractionation was carried out to purify the active compounds present in P515 and P605 producer strains.

### TZAs A and B inhibit R388 conjugation

**Fig 1:**
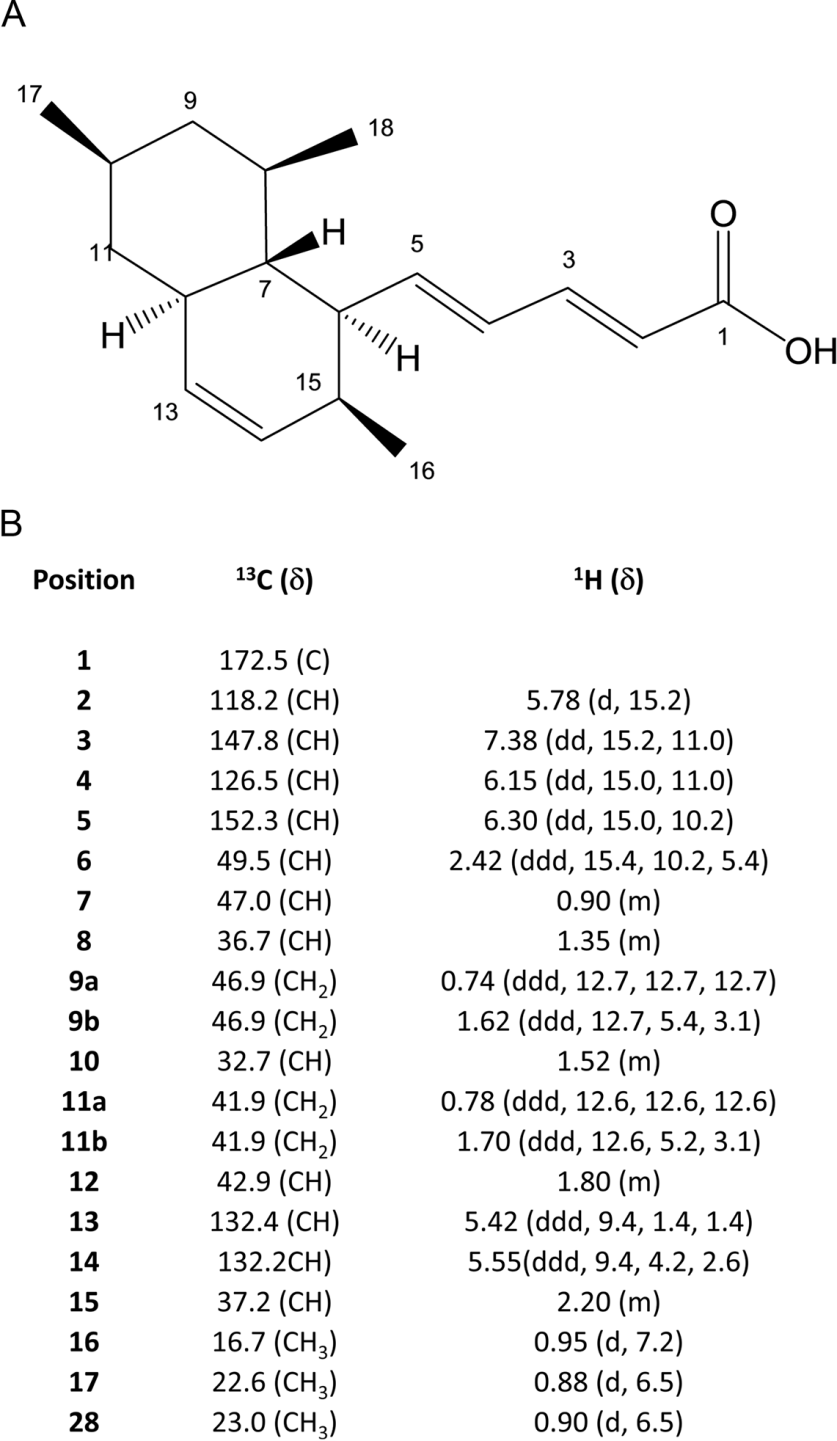
Structural elucidation of TZA-B. (A) Chemical structure of TZA-B, indicating carbon positions. (B) ^1^H and ^13^C NMR spectral data of TZA-B [δ (ppm), J_HH_ (Hz); CDCl_3_].

**Fig 2:**
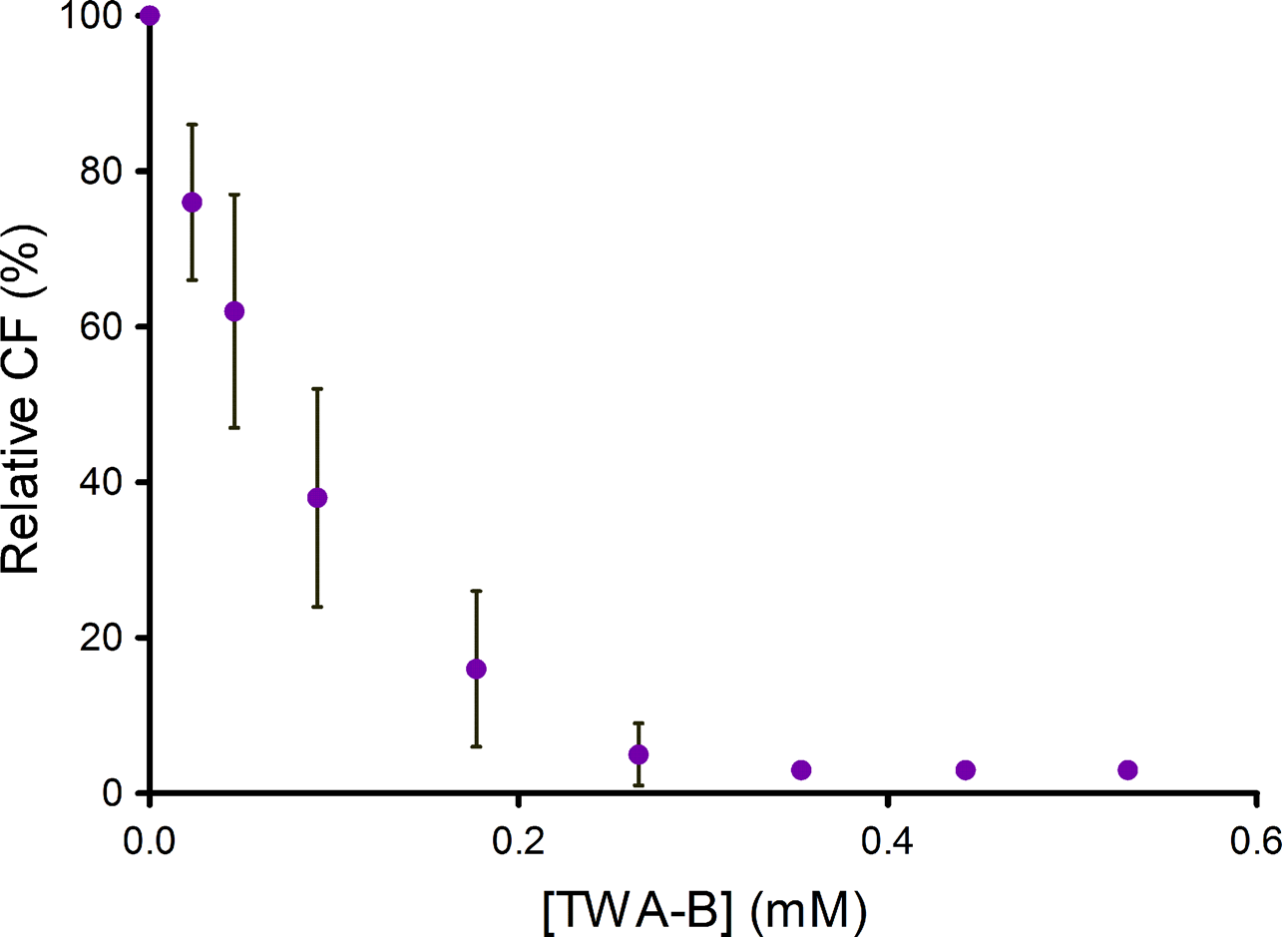
Conjugation frequency (CF) in the presence of increasing concentrations of TZA-B. Values represent the mean CF ± SD of at least four independent experiments, measured by fluorescence-based HTC assay and relative to positive control in the absence of COINs (100%).

Fractionation of extracts obtained from P515 and P605 producer strains was guided by a HTC assay based on fluorescence emission by transconjugant cells [10]. Re-fermentation of the P515-producing strain did not allow the purification of any active compound. Guided fractionation of P605 allowed the purification of one active compound, whose structure was elucidated by nuclear magnetic resonance (Fig 1). The new COIN was identified as TZA-B, a polyketide previously described as inhibitor of superoxide anion production from *Penicillium citrinum* [23, 24]. Dose/response analysis of TZA-B was also performed by fluorescence-based HTC assay. As a result, 0.4 mM TZA-B was found to inhibit R388 conjugation to 2% (Fig 2), as confirmed by plate-conjugation assay (2 ± 2%).

**Fig 3:**
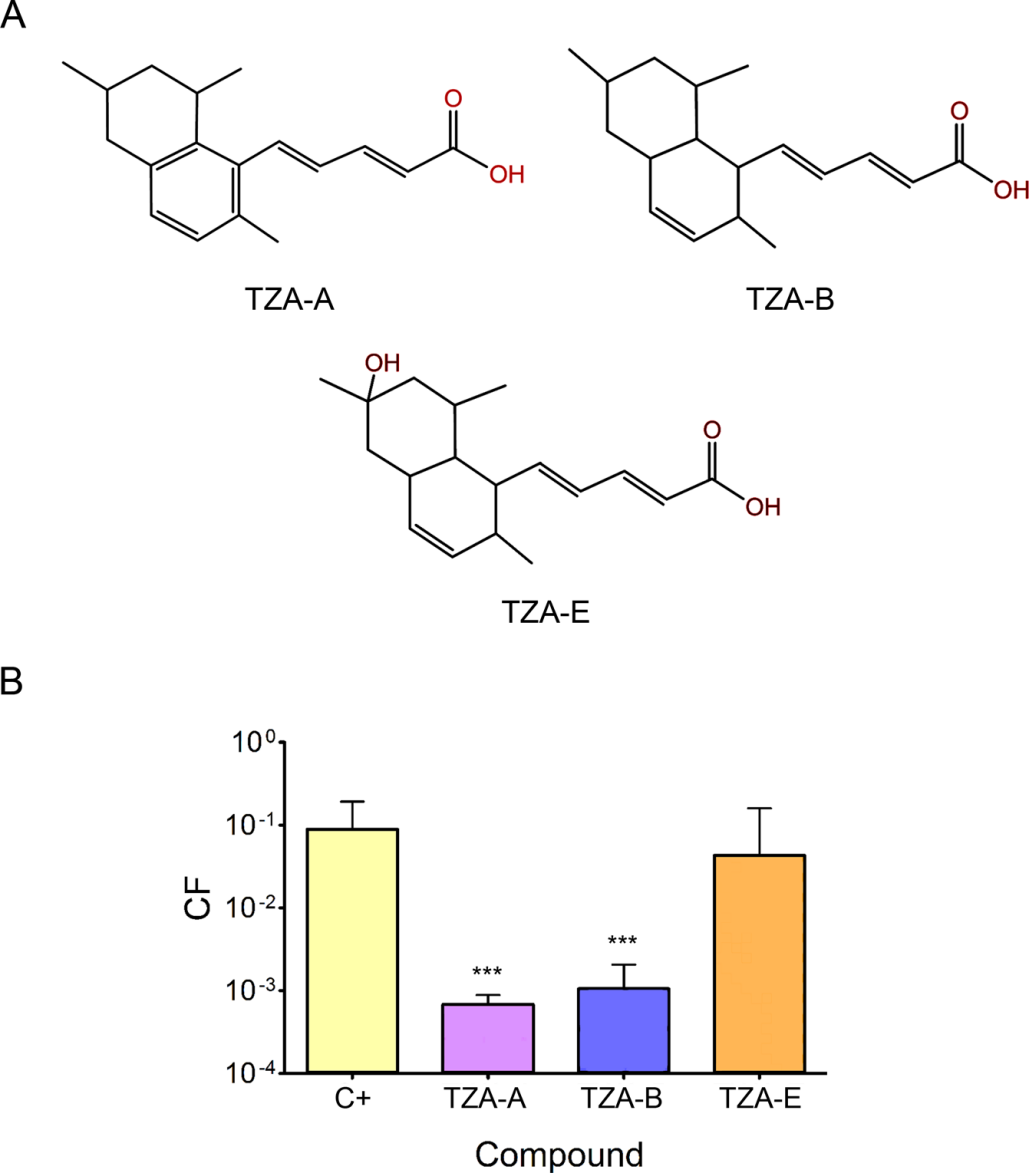
TZAs A, B and E structure and activity. (A) Chemical structure of TZAs A, B, and E. (B) CF of plasmid R388, measured by plate-conjugation assay and represented in logarithmic scale in the presence of 1 mM TZAs A, B, or E. C+, control in the absence of added compound. Bars represent the mean CF + SD of at least three independent experiments (*** p < 0.001).

In the same way as TZA-B, two of its structural analogs, namely TZAs A and E (Fig 3A), are also inhibitors of superoxide anion production [23, 24]. They were also checked as possible COINs. While TZA-A inhibited R388 conjugation to levels similar to TZA-B, TZA-E, carrying an additional hydroxyl group in its chemical structure, did not show significant COIN activity (Fig 3B). Interestingly, TZA-A was present in one of the 9 hits selected in the primary HTC assay (S1 Fig), specifically AD0103 (S2 Fig), which contained 60% pure TZA-A.

### IncW and IncF conjugative plasmids, main targets

**Fig 4:**
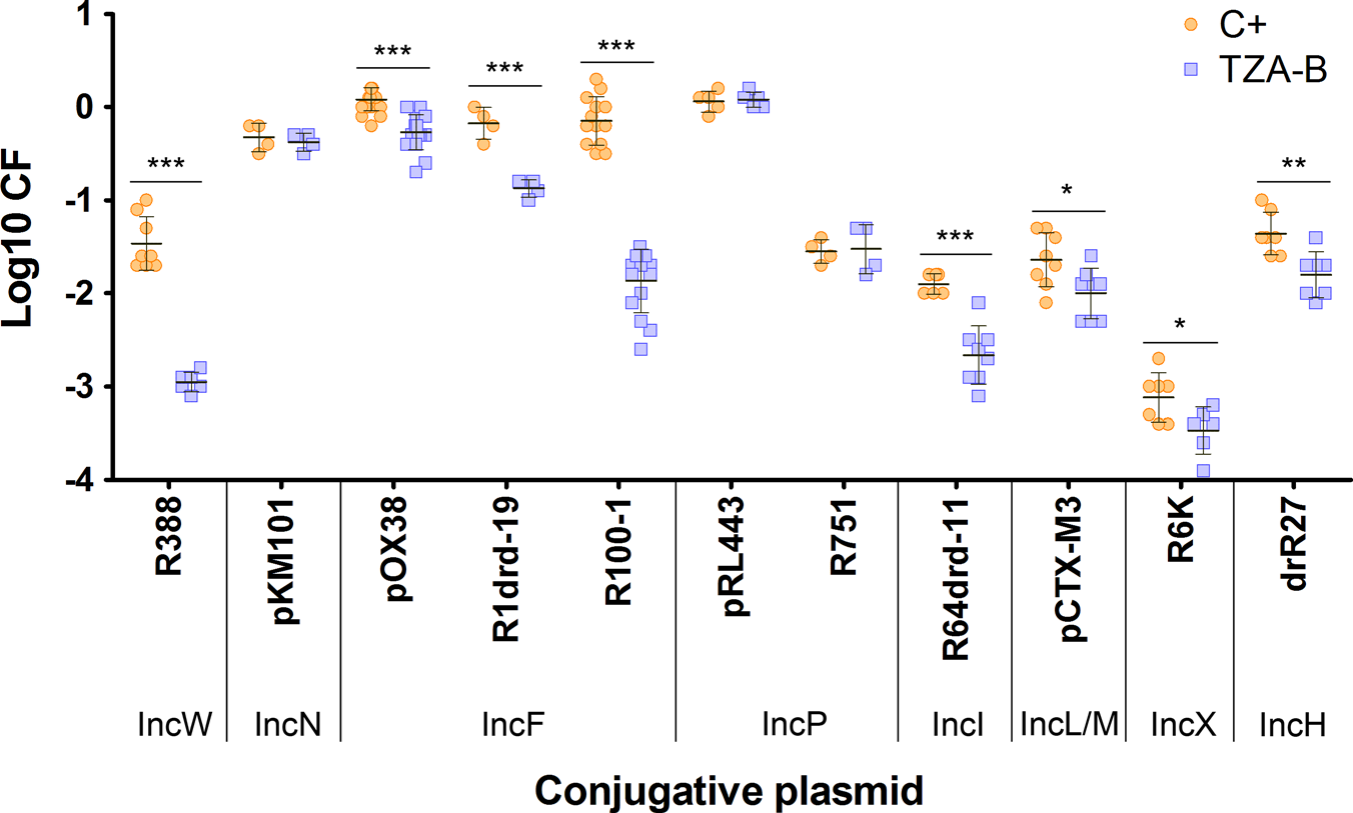
CF of prototype plasmids in the presence of TZA-B. CF in the presence of 0.4 mM TZA-B (TZA-B, blue squares) or in its absence (C+, orange circles) using a representative set of conjugative plasmids. Each point represents the result of one independent experiment in logarithmic scale measured by plate-conjugation assay. Horizontal and vertical bars represent the mean ± SD of each group of data (* p < 0.05, ** p < 0.01, *** p < 0.001). Inc, incompatibility group [25].

A collection of clinically representative conjugative plasmids found in Enterobacteriaceae was tested to investigate the range of TZA-B susceptible plasmids. Results are shown in Fig 4. Conjugation of the IncW plasmid R388 and the IncFII plasmid R100-1 was specially inhibited in the presence of TZA-B, almost 100-fold at 0.4 mM concentration. Besides, IncFI (pOX38), IncFII (R1drd19), IncI (R64drd11), IncL/M (pCTX-M3), IncX (R6K) and IncH (drR27) plasmids were also inhibited, although to a lesser extent (CF from 10 to 50%). Other plasmid groups, such as IncN and IncP, were not affected.

### TZA-B inhibits mobilization helped by IncW and IncF plasmids

**Fig 5:**
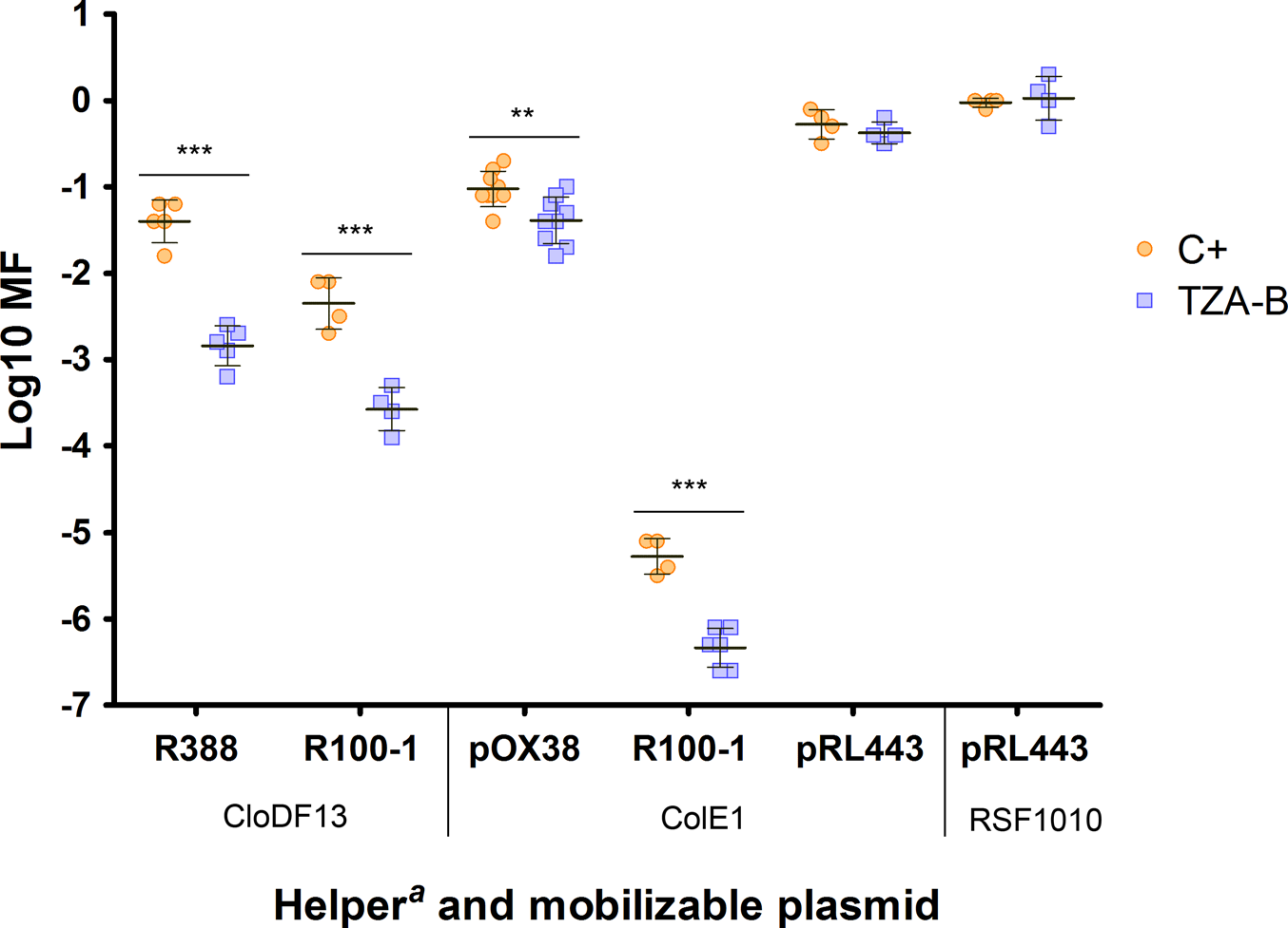
Mobilization frequency (MF) in the presence of TZA-B. MF of three mobilizable plasmids in the presence of 0.4 mM TZA-B (TZA-B, blue squares) or in its absence (C+, orange circles), using four different helper plasmids. Each point represents the result of one independent experiment in logarithmic scale measured by plate-conjugation assay. Horizontal and vertical bars represent the mean ± SD of each group of data (** p < 0.01, *** p < 0.001). ^*a*^ CFs of helper plasmids in the presence of mobilizable plasmids were similar to that obtained alone (Fig 4).

In addition to conjugative plasmids, mobilizable plasmids are also important carriers of AbR genes. For mobilization, they need the MPF system of a conjugative plasmid present in the donor cell, and even its coupling protein in some cases (ColE1 and RSF1010). To find out which mobilizable plasmids were affected by TZA-B, mobilization of ColE1, RSF1010 and CloDF13 was analyzed in the presence of different helper plasmids. As shown in Fig 5, mobilization of plasmids CloDF13 (which encodes its own coupling protein) or ColE1 was affected when the helper plasmid used was itself susceptible to TZA-B (R388, pOX38 or R100-1). On the other hand, mobilization of ColE1 and RSF1010 plasmids helped by the COIN-resistant plasmid pRL443, was unaffected.

### Toxicity of natural and chemically synthesized COINs

Toxicity of COINs must be analyzed to select compounds that affect minimally the biodiversity of the targeted ecosystem. It was previously shown that concentrations around the COIN-IC_50_ dose are enough for a compound to prevent the spread of a conjugative plasmid in a bacterial population [10]. Thus, in order to assess the toxicity levels of different COINs, we must determine whether levels below the COIN-IC_50_ exert toxic effects in higher organisms or microbial species. For this purpose, we compared cytotoxic, antibacterial and antifungal activities of the various types of COINs discovered to date, using a variety of human cell lines, bacterial and fungal strains. As shown in Table 1, toxicity values (toxic-IC_50_) of all COINs on human cell lines was around 100 μM. Antibacterial and antifungal activities were more variable. Synthetic 2-ODA was bactericidal (toxic-IC_90_) over 7 μM versus *Mycobacterium smegmatis*. 2-HDA was bactericidal versus *M*. *smegmatis* and fungicidal versus *Aspergillus nidulans* and *Candida albicans* at similar levels. On the contrary, TZA-A, TZA-B, oleic and linoleic acids showed significantly lower antibacterial and antifungal activities, their toxic-IC_90_ values ranging over 100 μM.

**Table 1:** Comparative analysis of COIN toxicity properties.

COIN	Toxic-IC_50_ (μM)					Toxic-IC_90_ (μM)			
	A549	HCT-116	PSN1	T98G	Fibroblasts	*Saur*	*Msme*	*Anid*	*Calb*
**TZA-A**	60	70	90	90	190	230	230	> 230	> 230
**TZA-B**	90	90	180	180	180	120	120	> 230	> 230
**2-HDA**	40	40	80	100	100	30	8	8	< 4
**2-ODA**	90	70	180	150	90	350	7	220	220
**Oleic acid**	40	180	80	180	350	> 420	> 420	> 220	> 220
**Linoleic acid**	90	180	90	180	300	> 360	360	> 360	> 360

Toxicity properties of TZA-A and TZA-B compared to previously described COINs [6, 10]. Inhibitory concentrations for cytotoxic (toxic-IC_50_), antibacterial and antifungal activities (toxic-IC_90_) using different human cell lines, bacteria and fungus species (mean value of three independent experiments). Saur, *Staphylococcus aureus*; Msme, *M*. *smegmatis*; Anid, *A*. *nidulans*; Calb, *C*. *albicans*.

## Discussion

COIN application in clinical and environmental settings demands non-toxic, easy to obtain, chemically and biologically stable molecules. COINs discovered to date have limitations that deviate from ideality, such as obtainability, stability or toxicity [6, 10]. For that reason, a collection of natural compounds extracted from marine microorganisms was screened to find compounds suitable for environmental use. Using AQUAc, a collection of partially purified natural compounds, two new COINs, TZA-A and TZA-B, were discovered (Fig 3). Their potency (Fig 2) was similar to that of previously identified uFAs (oleic and linoleic acids) [6] and of the chemically synthesized 2-HDA [10].

TZAs are fungal polyketides with chemical structures more complex than previously described COINs [6, 10]. They are carboxylic acids containing two aromatic rings at the end of an unsaturated aliphatic chain. As a consequence, they belong to the same group as previously reported inhibitors. The independent isolation of these compounds confirms the essentiality of these two chemical characteristics (a carboxylic group and a long, unsaturated aliphatic chain) for COIN activity. Interestingly, the TZA variant TZA-E, which contains an additional hydroxyl group distal to the carboxylic acid in its chemical structure (Fig 3A), was inactive (Fig 3B). It thus seems that a substantial hydrophobic moiety is important for COIN function, a characteristic that is functionally broken by a distant single hydroxyl group in the bulky TZA-E.

In addition to potency and structural similarities, the shared spectra of plasmids affected by the action of TZAs and previously analyzed COINS points to a common mechanism of inhibition. IncW and IncF conjugative plasmids, as well as their mobilizable plasmids, represent the main targets of the COINs described here (Figs 4 and 5) as well as in previous publications [6, 10]. These results suggest a shared target in conjugation and mobilization, probably being part of the MPF system of affected conjugative plasmids, also used for transfer of mobilizable plasmids.

TZAs were previously reported to inhibit superoxide anion production [23, 24], nitric oxide production and protein tyrosine phosphatase 1B activity in inflammatory cells [26]. In addition, two recent studies analyzed antimicrobial and cytotoxic effects of these fungal polyketides. TZA-A was found to inhibit conidial germination of the rice blast fungus *Magnaporthe oryzae* (toxic-IC_50_ = 37 μM), and showed weak activity against the Gram-positive bacteria *Brevibacillus brevis*, the fungi *Mucor miehei* and *Paecilomyces variotii*, and HeLaS3 cells at a concentration of 185 μM. Germination of the grey mold *Botrytis cinerea* and the potato blight caused by the oomycete *Phytophthora infestans* were not affected at comparable concentrations [27]. In an independent work [28], the antimicrobial activity of TZA-B against *S*. *aureus*, *Salmonella sp*., *Klebsiella pneumoniae*, *E*. *coli*, *Bacillus cereus*, *Proteus mirabilis*, *Enterococcus faecalis*, and *C*. *albicans* showed no effect below 364 μM COIN concentration. Moreover, leukemic and lymphoblastic cell lines (K562, U937, Jurkat and Raji) showed no response at 100 μM. These data, together with our results (Table 1), situate the TZAs, along with the previously identified oleic and linolenic acids [6], as the least toxic COINS identified so far. A comparison of toxicity values with the COIN potency of the different molecules tested, revealed that TZA-A, TZA-B, oleic and linoleic acid presented toxic-IC_50_ levels that were above their COIN threshold (COIN-IC_50_ ≈ 50 μM) (Fig 2) [6]. 2-HDA and 2-ODA were non-toxic at COIN concentrations in almost all human cell lines tested (COIN-IC_50_ ≈ 50 μM) [10], but exerted strong toxic effects in mycobacterial and/or fungal species (Table 1). Although TZA-B showed COIN activity at non-toxic concentrations, cytotoxic and COIN thresholds were too close. A key finding from this work is that toxicity and COIN activity do not necessarily correlate with each other, since TZA-B and 2-HDA presented similar COIN-IC_50_ concentrations, yet the later was more toxic to bacterial and fungal strains. This opens the possibility of further screening natural and synthetic derivatives with lower toxicity and enhanced COIN activity.

In summary, the COINs reported here and in previous work provide important ammunition in the search for optimal COINs. Their different characteristics make them applicable to different purposes. On the one hand, 2-HDA and 2-ODA are easily obtainable by chemical synthesis [14–16] and have provided important structural information [10]. Nevertheless, their antifungal [17, 18], antiprotozoal [14, 15], antimicrobial and cytotoxic activities [16, 19, 20], exclude their use in natural environments, where biodiversity must be maintained, and confine their use to academic setups. On the other hand, TZAs A and B (this work), as well as oleic and linoleic acids [6], are potentially more unstable, but they are natural compounds with reduced toxicity (Table 1), some of them being normal constituents of the human diet [29]. This makes them potential COINs for their use in natural environments, either in combination with effective antioxidants or through delivery vehicles with a protective atmosphere. In general, COINs show a shared and relatively broad range of affected plasmids, among them IncF plasmids, the most common AbR carriers in pathogenic *Enterobacteriaceae* [30]. Furthermore, non-toxic COINs could be used in ecological reservoirs of AbR genes, or as a combination treatment with antibiotics to prolong their useful lifetime, or even as virulence inhibitors for pathogens such as *Legionella*, *Helicobacter*, *Neisseria*, *Brucella* or *Bartonella*, which use secretion systems similar to conjugative systems.

## Materials and Methods

### Bacterial strains and plasmids

Derivatives of *E*. *coli* strain DH5α [31] containing either the conjugative plasmid pJC01 [10] or plasmids pSU2007::Tn*lux* and pUC18::*lacI*^*q*^ [6] were used as donor strains in fluorescence-based or luminescence-based HTC experiments, respectively. Rifampicin-resistant derivative *E*. *coli* CSH53 [32] was used as recipient strain in luminescence-based HTC assay and as pSU2007::Tn*lux* containing strain in control assays [6]. Streptomycin-resistant derivative *E*. *coli* BL21 (DE3) [33] was used as recipient strain expressing T7 RNA polymerase in fluorescence-based HTC assay [10]. *E*. *coli* DH5α [31] containing different conjugative and mobilizable plasmids (S2 Table) and a rifampicin-resistant derivative of *E*. *coli* MDS52 [34] were used as donor and recipient strains respectively in plate-conjugation assays.

### Reagents

When appropriate, antibiotics (Apollo) were added at the following concentrations: ampicillin sodium salt (Ap; 100 μg/ml), chloramphenicol (Cm; 25 μg/ml), nalidixic acid (Nx; 20 μg/ml), rifampicin (Rif; 50 μg/ml), streptomycin (Sm; 300 μg/ml), tetracycline (Tc; 10 μg/ml) and trimethoprim (Tp; 10 μg/ml). Oleic and linoleic acids (Sigma-Aldrich) were used as control COINs, DMSO (Sigma-Aldrich) was used as solvent and IPTG (Sigma-Aldrich) as T7 RNA polymerase inductor. Bacterial cultures were set up in LB-broth and LB-agar (Pronadisa). M9 broth (Sigma-Aldrich) were used to resuspend bacteria after mating and perform serial dilutions.

### Isolation of TZA-B

TZA-B producer *Penicillium* sp. strain CECT 20935, isolated from a *Porifera* sp. collected in Guatemala and grown in potato dextrose agar plates (Pronadisa), was used to inoculate 40 ml of potato dextrose broth (Pronadisa). This first inoculum was grown for 3 days at 24°C and 200 rpm. Then, 15 ml were added to 250 ml of the same media and cultured for 7 days at 24°C and 200 rpm. Fermentation broth (4 l) was filtered off with dicalite^®^ (Dicalite Europe) and the mycelial cake was extracted twice by adding 1.5 l of a mixture of EtOAc/MeOH 3:1 and soaking for 1 h. The organic solvent was filtered off and the pellet dried under reduced pressure. Dried extracts (2.8 g) were fractionated by vacuum flash chromatography using a stepwise gradient of Hexane/EtOAc/MeOH. Fractions containing TZA-B (eluted with Hexane/EtOAc 2:8) were applied to a silica gel column and flash-chromatographed by elution with a Hexane/EtOAc gradient. Fractions eluted with Hexane/EtOAc 75:25, afforded 105 mg of 93% pure TZA-B.

### Structural elucidation of TZA-B

TZA-B has a maximum UV absorption at 300 nm. The molecular formula was determined to be C_18_H_26_O_2_ based on the MS (*m/z* 274.3) and NMR spectral data. Extensive NMR experiments (^1^H NMR, ^13^CNMR, ^1^H-^1^H COSY, gHSQC, gHMBC and NOESY) indicate that TZA-B has three methyl groups, two methylenes, twelve methines (six of them olefinic), one quaternary carbon and one exchangeable proton. These data were identical with those for TZA-B, previously reported in the literature [23]. ^1^H-NMR and ^13^C-NMR data (Fig 1) were recorded on a Varian “Mercury 400” spectrometer (Agilent Technologies) at 400 and 100 MHz, respectively. gHMQC and gHMBC experiments were carried out using an inverse resonance probe. Chemical shifts are reported in ppm relative to solvent (CDCl_3_ δ_H_ 7.24, δ_C_ 77.0). MS data were recorded on an Agilent/HP 1100 Series Simple Quad Mass Spectrometer (Agilent Technologies), using both, ESI (+) y (-) and APCI (+) y (-) ionization sources.

### HTC screening

A luminescence-based HTC assay was performed as previously described [6]. Briefly, a *lux* operon under the control of a *lac* promoter encoded in the R388 derivative pSU2007::Tn*lux* is repressed in donor cells by the LacI^q^ repressor carried in the co-resident non-mobilizable multi-copy plasmid pUC18::*lacI*^*q*^. Upon conjugation, pSU2007::Tn*lux* but not pUC18::*lacI*^*q*^ is transferred to recipient cells, where light is produced. Absolute luminescence emitted by transconjugant cells was then measured and normalized to the mean value of the corresponding plate. Control assays to discard non-specific compounds were carried out by growing a pSU2007::Tn*lux* containing strain without plasmid pUC18::*lacI*^*q*^ and measuring light production. Similarly, HTC assay based on the emission of fluorescence employed plasmid pJC01 as test plasmid [10]. In donor cells, the *gfp* gene present in this R388 derivative is not expressed, due to the inactivity of its T7 promoter. When pJC01 plasmid is transferred to the recipient strain, which carries T7 RNA polymerase, GFP is produced. CF was estimated as the ratio of absolute fluorescence emitted by transconjugant cells and OD_600_ as a measurement of the total number of cells. Relative CF in the presence of a compound was thus determined as a fraction of the CF in the absence of it (adding the same volume of solvent).

### Plate-conjugation assay

For the plate-mating procedure, a 200 μl mixture of equal volumes of donor and recipient cultures previously washed, both in stationary phase, was centrifuged and resuspended in 15 μl LB-broth. 5 μl of this mixture were placed on top of 96-well microtiter plate wells containing 150 μl LB-agar (± COINs) and conjugation was allowed to proceed, in general, for 1 h at 37°C. The temperature-sensitive IncH plasmid drR27 was allowed to conjugate for 2 h at 25°C [35]. Bacteria were then resuspended in 150 μl M9 broth and corresponding dilutions were plated on selective media. CF was calculated as the number of transconjugant cells per donor, whereas MF was calculated as the number of cells receiving the mobilizable plasmid per donor. Since this type of frequency data were log-normally distributed, means are calculated using decimal logarithms of data. Relative CF or MF in the presence of a compound was determined as a fraction of the CF of MF in the absence of it (adding the same volume of solvent).

### Toxicity assays

Cell culture cytotoxicity assays were performed as described [36, 37] using human foreskin fibroblasts ATCC SCRC-1041 [38], lung carcinoma cells A549 [39], colorectal carcinoma cells HCT-116 [40], pancreatic adenocarcinoma cells PNS1 [41] or glioblastoma multiforme cells T98G [42]. Antibacterial activity was determined using a conventional microtiter broth-dilution technique [43] for two reference strains, *S*. *aureus* CECT 794 and *M*. *smegmatis* DSMZ 43756. Antifungal activity was measured using the reference method antifungal broth dilution susceptibility test (National Committee for Clinical Laboratory Standars) against two species: *A*. *nidulans* (Microorganisms Collection of Biomar Microbial Technologies) and *C*. *albicans* CECT 1394.

### Statistical analysis

Mean comparison between two different conditions was carried out by using t test tool from GraphPad Prism^®^ (v 5.0) biostatistics software (San Diego, CA).

## Supporting Information

S1 Fig Point cloud representation obtained from AQUAc HTC screening. Absolute luminescence emitted by transconjugant cells was measured in arbitrary light units (A. L. U.) and normalized to the mean value of the corresponding plate (100%). Each point represents the mean of two independent experiments obtained by luminescence-based HTC assay in the presence of bactericidal or non-bactericidal compounds (220 ng/ml or 11 μg/ml, respectively). A relative luminescence cutoff of 10% was arbitrarily established (red) to select the most active compounds. Oleic and linoleic acids (green) were used at 1 mM concentration as control COINs.
(TIF)

S2 Fig Kinetic luminescence assay of selected hits from AQUAc screening. *E*. *coli* CSH53 containing pSU2007::Tn*lux* (but not pUC18::*lacI*^*q*^) was cultured overnight, diluted until OD_600_ = 0.1 and grown for 2 h in the absence (C+) or the presence of each potential inhibitor (50 μg/ml). The figure shows the kinetics of light emission, measured every 5 min and represented over time.
(TIF)

S1 Table Potency of AQUAc selected hits. CF in the presence of selected hits from AQUAc screening. Absolute luminescence emitted by transconjugant cells was measured in A.L.U. and relativized to the control in the absence of added COINs (100%). Each value represents the mean of two independent experiments obtained by luminescence-based HTC assay in the presence of the given concentrations of selected hits. The hyphen represents no data for that point.
(DOCX)

S2 Table Conjugative and mobilizable plasmids used. (DOCX)
