# Supplementary figures and images for: Tanzawaic Acids, a Chemically Novel Set of Bacterial Conjugation Inhibitors
Source: PLoS One. 2016 Jan 26;11(1):e0148098. doi: 10.1371/journal.pone.0148098 (PMC4727781; doi:10.1371/journal.pone.0148098)

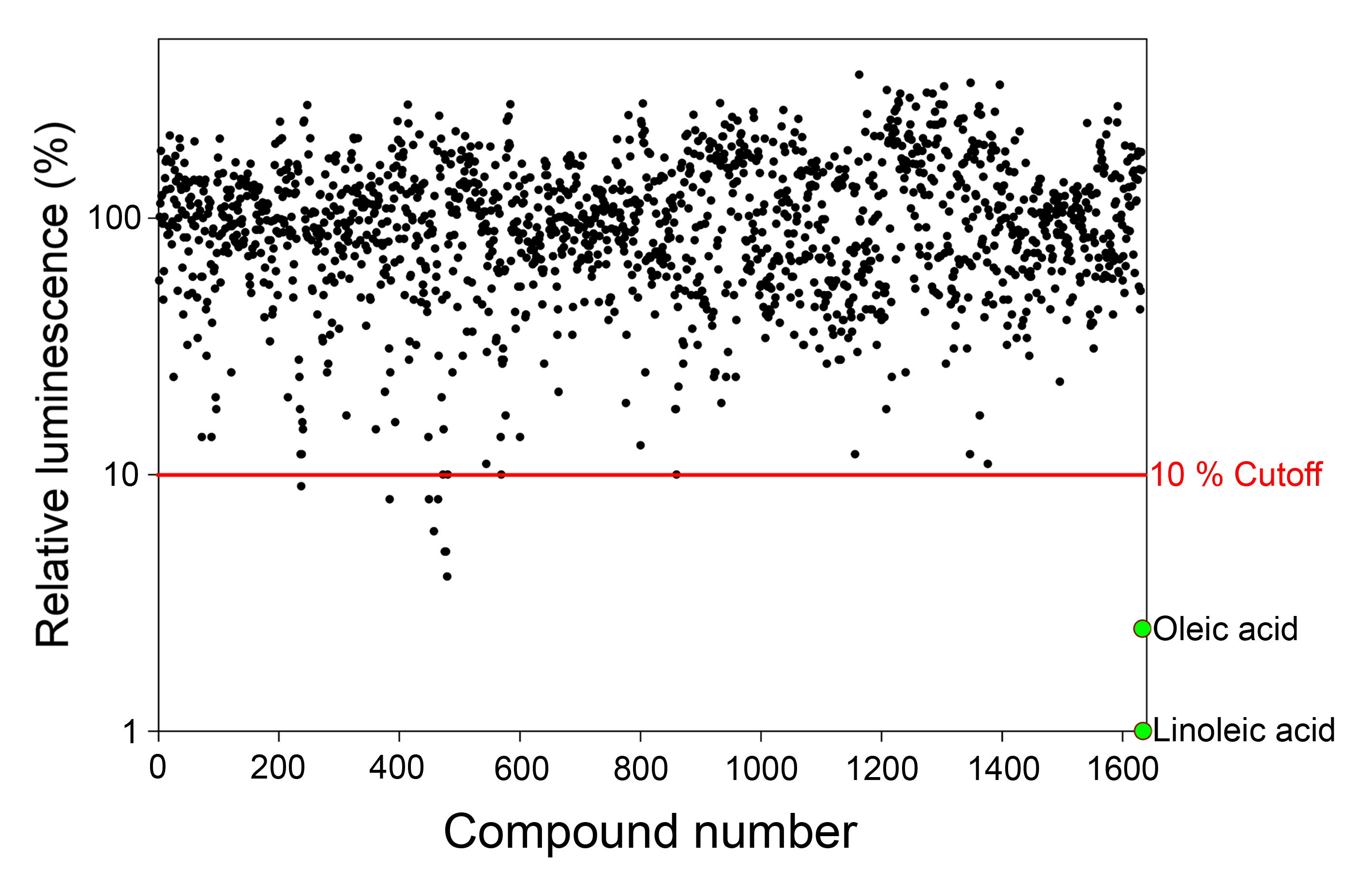

Supplement: S1 Fig — Absolute luminescence emitted by transconjugant cells was measured in arbitrary light units (A. L. U.) and normalized to the mean value of the corresponding plate (100%). Each point represents the mean of two independent experiments obtained by luminescence-based HTC assay in the presence of bactericidal or non-bactericidal compounds (220 ng/ml or 11 μg/ml, respectively). A relative luminescence cutoff of 10% was arbitrarily established (red) to select the most active compounds. Oleic and linoleic acids (green) were used at 1 mM concentration as control COINs. (TIF) [file pone.0148098.s001.tif]

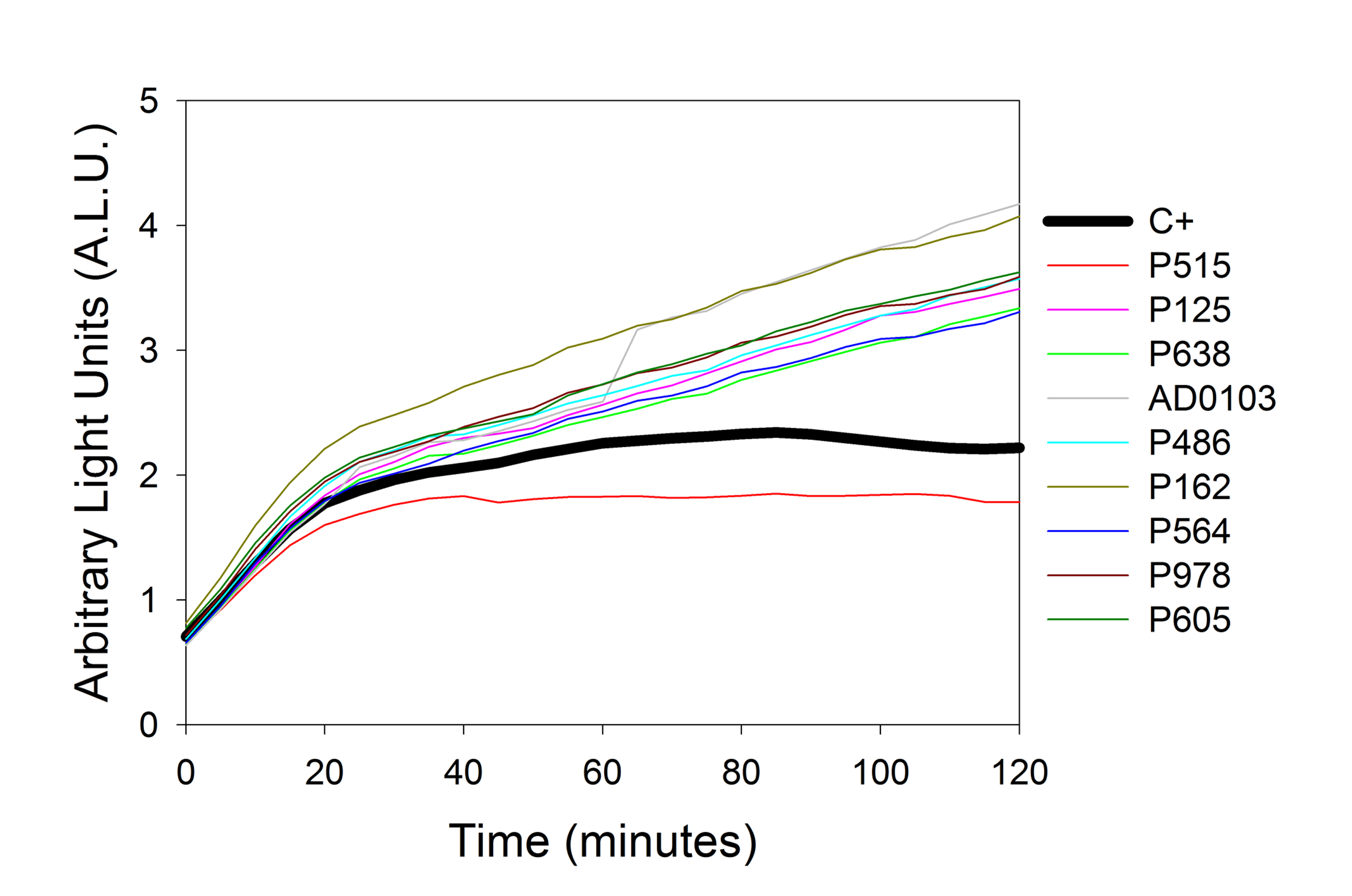

Supplement: S2 Fig — E. coli CSH53 containing pSU2007::Tnlux (but not pUC18::lacIq) was cultured overnight, diluted until OD600 = 0.1 and grown for 2 h in the absence (C+) or the presence of each potential inhibitor (50 μg/ml). The figure shows the kinetics of light emission, measured every 5 min and represented over time. (TIF) [file pone.0148098.s002.tif]
