# Supplementary material for: Tanzawaic Acids, a Chemically Novel Set of Bacterial Conjugation Inhibitors
Source: PLoS One. 2016 Jan 26;11(1):e0148098. doi: 10.1371/journal.pone.0148098 (PMC4727781; doi:10.1371/journal.pone.0148098)
Supplement: S1 Table — CF in the presence of selected hits from AQUAc screening. Absolute luminescence emitted by transconjugant cells was measured in A.L.U. and relativized to the control in the absence of added COINs (100%). Each value represents the mean of two independent experiments obtained by luminescence-based HTC assay in the presence of the given concentrations of selected hits. The hyphen represents no data for that point. (DOCX) [file pone.0148098.s003.docx]

**S1 Table. Potency of AQUAc selected hits.**

| **Hit** | **CF (%)** | | | |
| --- | --- | --- | --- | --- |
|  | **5 μg/ml** | **10 μg/ml** | **25 μg/ml** | **50 μg/ml** |
| **P515** | 1 | 1 | 1 | 1 |
| **P125** | 55 | 22 | 15 | 8 |
| **P638** | 94 | 75 | 28 | 25 |
| **AD0103** | 109 | 91 | 42 | 19 |
| **P486** | - | 17 | 26 | 7 |
| **P162** | 17 | 23 | 23 | 6 |
| **P564** | 110 | 83 | 98 | 22 |
| **P978** | 22 | 26 | 23 | 8 |
| **P605** | 5 | 6 | 6 | 2 |

CF in the presence of selected hits from AQUAc screening. Absolute luminescence emitted by transconjugant cells was measured in A.L.U. and relativized to the control in the absence of added COINs (100 %). Each value represents the mean of two independent experiments obtained by luminescence-based HTC assay in the presence of the given concentrations of selected hits. The hyphen represents no data for that point.
