# Supplementary material for: Tanzawaic Acids, a Chemically Novel Set of Bacterial Conjugation Inhibitors
Source: PLoS One. 2016 Jan 26;11(1):e0148098. doi: 10.1371/journal.pone.0148098 (PMC4727781; doi:10.1371/journal.pone.0148098)
Supplement: S2 Table — (DOCX) [file pone.0148098.s004.docx]

**S2 Table. Conjugative and mobilizable plasmids used.**

| Plasmid | Inc*^a^* | MOB*^b^* | MPF*^c^* | Reference |
| --- | --- | --- | --- | --- |
| R388 | W | F11 | T | [[1](#_ENREF_1)] |
| pKM101 | N | F11 | T | [[2](#_ENREF_2)] |
| pOX38 | FI | F12 | F | [[3](#_ENREF_3)] |
| R1drd-19 | FII | F12 | F | [[4](#_ENREF_4)] |
| R100-1 | FII | F12 | F | [[5](#_ENREF_5)] |
| pRL443 | P1α | P11 | T | [[6](#_ENREF_6)] |
| R751 | P1β | P11 | T | [[7](#_ENREF_7)] |
| R64drd-11 | I1α | P12 | I | [[8](#_ENREF_8)] |
| pCTX-M3 | L/M | P131 | I | [[9](#_ENREF_9)] |
| R6K | X2 | P3 | T | [[10](#_ENREF_10)] |
| drR27 | HI1 | H11 | F | [[11](#_ENREF_11)] |
| CloDF13 | ColE | C11 | - | [[12](#_ENREF_12)] |
| ColE1 | ColE | P5 | - | [[13](#_ENREF_13)] |
| RSF1010 | Q1 | Q11 | - | [[14](#_ENREF_14)] |

*^a^* Inc, incompatibility group [[15](#_ENREF_15)].

*^b^* MOB, MOB group [[16](#_ENREF_16)].

*^c^* MPF, mating pair formation type [[17](#_ENREF_17)]. The hyphen represents the absence of MPF in mobilizable plasmids.

**References**

1. Datta N, Hedges RW. Trimethoprim resistance conferred by W plasmids in Enterobacteriaceae. Journal of general microbiology. 1972;72(2):349-55. PubMed PMID: 4562309.

2. Langer PJ, Shanabruch WG, Walker GC. Functional organization of plasmid pKM101. Journal of bacteriology. 1981;145(3):1310-6. PubMed PMID: 6259131.

3. Chandler M, Galas DJ. Cointegrate formation mediated by Tn9. II. Activity of IS1 is modulated by external DNA sequences. Journal of molecular biology. 1983;170(1):61-91. PubMed PMID: 6313938.

4. Meynell E, Datta N. Mutant drug resistant factors of high transmissibility. Nature. 1967;214(5091):885-7. PubMed PMID: 6054969.

5. Yoshioka Y, Ohtsubo H, Ohtsubo E. Repressor gene finO in plasmids R100 and F: constitutive transfer of plasmid F is caused by insertion of IS3 into F finO. Journal of bacteriology. 1987;169(2):619-23. PubMed PMID: 3027040.

6. Elhai J, Vepritskiy A, Muro-Pastor AM, Flores E, Wolk CP. Reduction of conjugal transfer efficiency by three restriction activities of Anabaena sp. strain PCC 7120. Journal of bacteriology. 1997;179(6):1998-2005. PubMed PMID: 9068647; PubMed Central PMCID: PMC178925.

7. Thorsted PB, Macartney DP, Akhtar P, Haines AS, Ali N, Davidson P, et al. Complete sequence of the IncPbeta plasmid R751: implications for evolution and organisation of the IncP backbone. Journal of molecular biology. 1998;282(5):969-90. PubMed PMID: 9753548.

8. Komano T, Funayama N, Kim SR, Nisioka T. Transfer region of IncI1 plasmid R64 and role of shufflon in R64 transfer. Journal of bacteriology. 1990;172(5):2230-5. PubMed PMID: 1970558.

9. Golebiewski M, Kern-Zdanowicz I, Zienkiewicz M, Adamczyk M, Zylinska J, Baraniak A, et al. Complete nucleotide sequence of the pCTX-M3 plasmid and its involvement in spread of the extended-spectrum beta-lactamase gene blaCTX-M-3. Antimicrobial agents and chemotherapy. 2007;51(11):3789-95. PubMed PMID: 17698626.

10. Kolter R, Helinski DR. Construction of plasmid R6K derivatives in vitro: characterization of the R6K replication region. Plasmid. 1978;1(4):571-80. PubMed PMID: 372982.

11. Whelan KF, Maher D, Colleran E, Taylor DE. Genetic and nucleotide sequence analysis of the gene htdA, which regulates conjugal transfer of IncHI plasmids. Journal of bacteriology. 1994;176(8):2242-51. Epub 1994/04/01. PubMed PMID: 7908903; PubMed Central PMCID: PMC205345.

12. van Putten AJ, Jochems GJ, de Lang R, Nijkamp HJ. Structure and nucleotide sequence of the region encoding the mobilization proteins of plasmid CloDF13. Gene. 1987;51(2-3):171-8. PubMed PMID: 3596243.

13. van Rensburg AJ, Hugo N. Characterization of DNA of colicinogenic factor E1 in a providence strain. Journal of general microbiology. 1969;58(3):421-2. PubMed PMID: 4904094.

14. Derbyshire KM, Hatfull G, Willetts N. Mobilization of the non-conjugative plasmid RSF1010: a genetic and DNA sequence analysis of the mobilization region. Mol Gen Genet. 1987;206(1):161-8. PubMed PMID: 3033438.

15. Taylor DE, Gibreel A, Lawley TD, Tracz DM. Antibiotic resistance plasmids. In: Funnel BE, Phillips GJ, editors. Plasmid Biology. Washington, DC: ASM Press; 2004. p. 473-91.

16. Garcillan-Barcia MP, Francia MV, de la Cruz F. The diversity of conjugative relaxases and its application in plasmid classification. FEMS microbiology reviews. 2009;33(3):657-87. PubMed PMID: 19396961.

17. Guglielmini J, Quintais L, Garcillan-Barcia MP, de la Cruz F, Rocha EP. The repertoire of ICE in prokaryotes underscores the unity, diversity, and ubiquity of conjugation. PLoS genetics. 2011;7(8):e1002222. PubMed PMID: 21876676.
